# Factors Related to Long-Term Survival in Patients Affected by Well-Differentiated Endocrine Tumors of the Pancreas

**DOI:** 10.5402/2012/389385

**Published:** 2012-07-02

**Authors:** Riccardo Casadei, Claudio Ricci, Paola Tomassetti, Davide Campana, Francesco Minni

**Affiliations:** ^1^Department of Surgery, S.Orsola-Malpighi Hospital, Alma Mater Studiorum, University of Bologna, 40138 Bologna, Italy; ^2^Department of Internal Medicine, S.Orsola-Malpighi Hospital, Alma Mater Studiorum, University of Bologna, 40138 Bologna, Italy

## Abstract

*Aim*. To identify factors related to survival in patients affected by well-differentiated PETs (benign, uncertain behavior, and carcinoma) who underwent R0 pancreatic resection. *Methods*. Retrospective study of 74 consecutive patients followed up from January 1980 to December 2011. Prognostic factors were sex, age, type of tumor, presence of symptoms, type of surgical procedure, size of tumor, lymph nodes status, WHO classification, and TNM stage. Overall survival was evaluated using the Kaplan-Meier method. Cox regression analyses were used to identify the factors associated with prognosis in univariate and multivariate analysis. *Results*. The mean follow-up of all the patients was 106 ± 89 months. The 5–10-year long-term survival was 90.9% and 79.1%, respectively. At univariate analysis, patient age <55 years was significantly related to a better long-term survival compared to patients age ≥55 years (307 ± 15 months versus 192 ± 25 months; *P* = 0.010). Multivariate analysis showed that female gender (*P* = 0.006), patients without comorbidities (*P* = 0.033), and patients affected by well-differentiated benign pancreatic endocrine tumors (*P* = 0.008 and *P* = 0.002 in relation to tumors with uncertain behavior and carcinomas, resp.) were factors significantly related to a better long-term survival. *Conclusions*. Patients factors were strongly related to a better long-term survival in patients observed. WHO classification is a very useful prognostic tool for well-differentiated PETs.

## 1. Introduction

Pancreatic endocrine tumors (PETs) are rare neoplasms with a poorly defined natural history that, in general, have a more indolent tumor biology with better long-term survival rates than tumors of the exocrine pancreas [1]. Surgery is generally considered the treatment of choice and, for patients with localized disease, it can be curative. The biological behavior of PETs is variable, necessitating close long-term follow-up. The World Health Organization (WHO) [2, 3] as well as tumor node metastasis (TNM) [4, 5] classifications recognized different groups of PETs with different biologic behaviors. Moreover, several authors [6–11] reported factors predicting survival after the resection of PETs. To our knowledge, there are no studies in the literature that reported prognostics factors related to long-term survival in patients with well-differentiated PETs. 

Thus, the present study was carried out in order to identify the factors related to long-term survival in patients affected by well-differentiated PETs (benign, uncertain behavior, and carcinoma) who underwent R0 pancreatic resection. 

## 2. Patients and Methods

A retrospective study of a prospective database of 74 consecutive patients who underwent R0 pancreatic resection for well-differentiated PETs (benign, uncertain behavior, and carcinoma) was conducted from January 1980 to December 2011. All the patients were followed up, and for each patient long-term overall survival (OS) was calculated from the date of surgery to the date of the death or last follow-up. December 2011 was the end of the follow-up period for surviving patients. Prognostic factors considered for each patient were sex, age (<55 or ≥55 years) [12] type of tumor (sporadic, functioning, nonfunctioning, and MEN-1), presence of symptoms, type of surgical procedure (typical or atypical resection), size of tumor (<2, 2–4, and >4 cm), lymph nodes status (N0, N1, and NX), WHO classification [2], and TNM stage [5]. No further therapy was initiated after R0 pancreatic resection even in cases with lymph nodes involvement. Follow-up examinations were conducted every 6 months for the first 2 years and every year subsequently with clinical examination, serum CgA, abdominal ultrasound, and computed tomography (CT) scan. Somatostatin receptor scintigraphic scanning (Octreoscan) of PET with Ga-Dotanoc was performed in selected cases in which there was the suspicious for recurrence of the disease. Surgical or medical therapy was performed in cases with recurrences.

## 3. Statistical Methods

Means, standard deviations, and frequencies were used to describe the data. OS was evaluated using the Kaplan-Meier method. Cox regression analyses were used to identify the factors associated with prognosis in univariate and multivariate analysis. Two-tailed *P* values less than 0.05 were considered statistically significant. All statistical analyses were carried out by running SPSS for Windows (version 13.0) on a personal computer. 

## 4. Results

Characteristics of the 74 patients affected by well-differentiated PETs who underwent R0 pancreatic resections are summarized in Table 1. There was a slight prevalence of women (52.7%) compared to men (47.3%); they had a mean age of 53 ± 14 years, and the major part of the patients were ≥55 years (52.7%). Twenty-seven (36.5%) patients had one or more comorbidities. Pancreatic endocrine tumors were more frequently symptomatic (76.3%) and nonfunctioning (54.1%). Functioning tumors were present in 34 (45.9%) cases: the major part (25 cases (33.8%)) were insulinomas; the others (9 cases (12.1%)) were 5 gastrinomas, 2 Vipomas, 1 glucagonoma, and 1 PPoma MEN-1 syndrome was rarely observed (7 cases (9.5%)). The tumors were usually small (size < 4 cm in 54 cases (72.9%)) and without lymph nodes involvement (66.2%). A typical resection was more frequently performed (73%) including left pancreatectomy (41 cases (55.4%)) pancreaticoduodenectomy (11 cases (14.9%)) and total pancreatectomy (2 cases (2.7%)). Atypical resection included 18 enucleoresection (24.3%) and 2 (2.7%) middle-pancreatectomy. Regarding the WHO classification of PETs, we observed, in the major part of the cases (45 cases (60.8%)), well-differentiated tumors, benign or with uncertain behavior. TNM stage III was the rarest (29.8%) stage with a prevalence of stage IIIb (23%).

**Figure 1:**
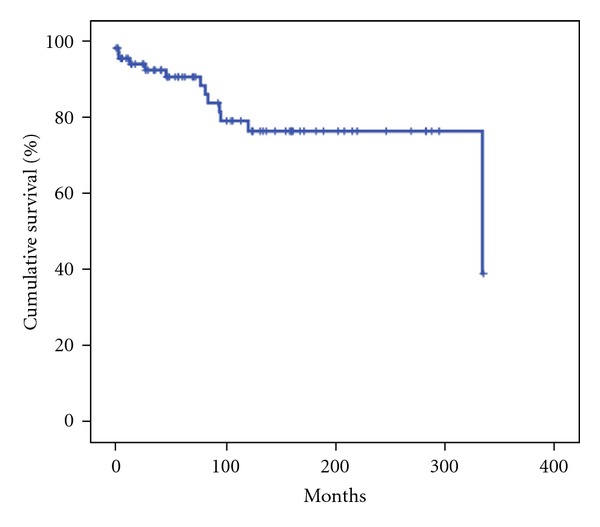
Long-term overall survival of the 74 patients who underwent R0 pancreatic resection for well-differentiated pancreatic endocrine tumors.

The mean follow-up of all the patients was 106 ± 89 months. During follow-up, 13 (17.6%) patients died after a mean time from surgery of 76 ± 87 months: 1 (1.3%) patient died in the postoperative period, 5 (6.8%) for disease progression, and 7 (9.5%) for causes not related to the tumor but to the patients. Patients deaths disease-related have a mean long-term survival of 68 ± 45 months. The mean long-term overall survival was 271 ± 16 months; 5–10-year long-term overall survival was 90.9% and 79.1%, respectively (Figure 1). Recurrence rate was 18.9% (14 cases), and in these cases the mean disease-free survival was 87 ±  56 months. In these cases, only in 1 (7.1%) a reoperation, consisting in a left pancreatectomy, was performed; the others were treated with somatostatin analogs.

At univariate analysis, symptoms, hormonal status, MEN-1 syndrome, size of tumor, lymph nodes status, type of resection, and TNM stage were not considered factors significantly related to long-term survival of the patients affected by well-differentiated PETs. Female gender presented a better long-term survival, but not statistically significant, compared to male (302 ± 19 versus 235 ± 27; *P* = 0.080) as well as patients without comorbidities compared to patients with comorbidities (290 ± 17 months versus 155 ± 21 months; *P* = 0.065). Regarding WHO classification, well-differentiated tumor-benign (WDT-B) showed a better, but not statistically significant, long-term survival than well-differentiated tumor-uncertain behavior (WDT-UB) and well-differentiated carcinoma (WDC) (280 ± 15 months versus 265 ± 34 and 235 ± 31 months, resp., *P* = 0.080). The only factor related to a significantly better long-term overall survival was the age: patients <55 years survived significantly more than patients ≥55 years (307 ± 15 months versus 192 ± 25 months; *P* = 0.010) (Table 2).

Multivariate analysis showed 3 factors significantly related to a better long-term overall survival: female gender (HR 0.2; C.I. 95% 0.4–0.6; *P* = 0.006), patients without comorbidities (patients with comorbidities  = HR 4.8; C.I. 95% 1.1–20.6; *P* = 0.033), and patients affected by a WDT-B, according to WHO classification (WDT-UB  = HR 25.3; C.I. 95% 2.3–280; *P* = 0.008, WDC  = HR 45.6; C.I. 95% 4.1–500; *P* = 0.002) (Table 3).

## 5. Discussion

Despite a considerable amount of research, our understanding of the natural history and predictors of survival of the PETs remains incomplete. However, it is well known that PETs are rather slow-growing tumors and they have a biological behavior less aggressive than pancreatic adenocarcinoma [1] with a 5-year survival ranging from 59.3% [12] to 79.5% [8]. Surgical R0 resection is the only potential curative treatment for patients affected by PETs. However, a small percentage of patients died during follow-up and the recurrence rate in these patients ranged from 24.5% to 36.3% [11]. Thus, the long-term survival seems to be related with prognostic factors: age, sex, functional status of the tumor, pancreatic resection, the presence or absence of cancer at the surgical margin, tumor location, tumor size, lymph nodes involvement, distant metastases, grade of the tumor, and TNM stage were the factors more frequently related to long-term survival in patients affected by PETs [6–12]. Finally, Bilimoria et al. [12] reported the prognostic score predicting survival after resection of PETs. 

WHO classification distinguished three groups of PETs: well-differentiated tumor (benign or with uncertain behavior), well-differentiated carcinoma, and poorly differentiated carcinoma. Several studies have suggested its prognostic significance and in particular they emphasize that well-differentiated tumors (benign, uncertain, and carcinoma) have a better long-term survival than poorly differentiated [7, 8]. To our knowledge, our study represents the first research of prognostic factors related to long-term survival in patients affected by well-differentiated tumors (benign, uncertain, and carcinoma) who underwent R0 pancreatic resection. First of all, it is to underline that the mean follow-up period was rather long (about 10 years) and the size sample was sufficient. Second, we reported that 5–10-year overall survival was excellent (90.9% and 79.1%, resp.), the recurrences rate was 18.9% with a mean time of 87 ± 56 months, and patients disease-related deaths rate was 6.8% with a mean long-term survival of 68 ± 45 months. These results suggest that these tumors are slow-growing neoplasms with indolent biologic behavior. Moreover, this result emphasizes that R0 pancreatic resection is the only change of cure for these tumors. At univariate analysis, the only factor related to a significantly better long-term overall survival was the patient age <55 years compared to patient age ≥55 years. Multivariate analysis, instead, showed that female gender, patients without comorbidities, and patients affected by a WDT-B were the prognostic factors related to a better long-term survival. Our results seem to suggest that patients factors (young age, female gender, and absence of comorbidities) represented a strong predictor of survival. This is probably due to the fact that young people, female gender, and patients without comorbidities have much more probabilities to live longer than elderly people, male gender, and patients without comorbidities. The fact that patients factors seem to be strongly related with long-term survival might reflect the favorable biological behavior of PETs. Finally, it is interesting to note that WHO classification showed a very strong prognostic significance because it is able to correlate the different groups of well-differentiated tumors (benign, uncertain behavior, and carcinoma) to a different prognosis.

In conclusion, our study suggests, first, that R0 pancreatic resection of well-differentiated PETs is the treatment of choice because of its good long-term results; second, mainly patients factors are related to a better long-term survival in patients affected by well-differentiated PETs who underwent R0 pancreatic resection, confirming the indolent biologic behavior of these tumors; third, WHO classification is a very useful prognostic tool for well-differentiated PETs.

## Figures and Tables

**Table 1:** Characteristics of the 74 patients who underwent R0 pancreatic resection for well-differentiated pancreatic endocrine tumors.

Factors	No. (%)
Sex	
M	35 (47.3)
F	39 (52.7)
Age	
<55 years	35 (47.3)
≥55–65** **years	39 (52.7)
Comorbidities	
None	47 (63.5)
One or more	27 (36.5)
Symptoms	
No	19 (25.7)
Yes	55 (74.3)
Hormonal status	
Nonfunctioning	40 (54.1)
Insulinoma	25 (33.8)
Others	9 (12.1)
MEN-1	
No	67 (90.5)
Yes	7 (9.5)
Size of tumors	
<2** **cm	30 (40.5)
2–4** **cm	24 (32.4)
>4 cm	20 (27.0)
Lymph node status	
N0	49 (66.2)
N1	17 (23.0)
Nx	8 (10.8)
Type of resection	
Typical	54 (73.0)
Atypical	20 (27.0)
WHO classification	
WDT-B	24 (32.4)
WDT-UB	21 (28.4)
WDEC	29 (39.2)
TNM stage^∗^	
I	26 (35.1)
II	26 (35.1)
III	22 (29.8)

WDT-B: well-differentiated tumor-benign; WDT-UB: well-differentiated tumor-uncertain behaviour; WDEC: well-differentiated carcinoma.

^∗^TNM-ENETS stage system modified according to Scarpa et al. [5].

**Table 2:** Univariate analysis of factors influencing overall survival in the 74 patients who underwent R0 pancreatic resection for well-differentiated pancreatic endocrine tumors.

Variables	Survival (months, mean ± SE)	*P* value
Sex		
M	235 ± 27	0.080
F	302 ± 19	
Age		
<55 years	307 ± 15	0.010
≥55 years	192 ± 25	
Comorbidity		
None	290 ± 17	0.065
One or more	155 ± 21	
Symptoms		
No	177 ± 22	0.794
Yes	273 ± 18	
Hormonal status		
Nonfunctioning	262 ± 25	0.430
Insulinoma	268 ± 17	
Others	221 ± 50	
MEN-1		
No	268 ± 17	0.646
Yes	253 ± 31	
Size of tumors		
<2 cm	236 ± 23	0.529
2–4 cm	300 ± 23	
>4 cm	247 ± 36	
Lymph node status		
N0	277 ± 21	0.966
N1	243 ± 38	
Nx	129 ± 15	
Type of resection		
Typical	272 ± 21	0.836
Atypical	270 ± 29	
WHO classification		
WDT-B	280 ± 15	0.080
WDT-UB	265 ± 34	
WDC	235 ± 31	
TNM stage^∗^		
I	241 ± 24	0.462
II	303 ± 25	
III	229 ± 35	

SE: standard error; WDT-B: well-differentiated tumor-benign; WDT-UB: well-differentiated tumor-uncertain behaviour; WDEC: well-differentiated carcinoma.

^∗^TNM-ENETS stage system modified according to Scarpa et al. [5].

**Table 3:** Multivariate analysis of factors influencing overall survival in the 74 patients who underwent R0 pancreatic resection for well-differentiated pancreatic endocrine tumors.

Variables	HR (C.I. 95%)	*P* value
Sex		
M	1.0	0.006
F	0.2 (0.4–0.6)	
Comorbidity		
None	1.0	0.033
One or more	4.8 (1.1–20.6)	
WHO classification		
WDT-B	1.0	
WDT-UB	25.3 (2.3–280)	0.008
WDEC	45.6 (4.1–500)	0.002

WDT-B: well-differentiated tumor-benign; WDT-UB: well-differentiated tumor-uncertain behaviour; WDEC: well-differentiated carcinomas.
